# Effect of metformin on pancreatic neuroendocrine tumors of multiple endocrine neoplasia type 1

**DOI:** 10.1111/jne.70166

**Published:** 2026-03-25

**Authors:** Serguei V. Kozlov, Sunita K. Agarwal, Theresa M. Guerin, Wendi Custer Lawrence, Laura L. Bassel, Akua Graf, Smita Jha, Lee S. Weinstein, Jenny E. Blau, Jaydira del Rivero

**Affiliations:** ^1^ Center for Advanced Preclinical Research, Frederick National Lab for Cancer Research, Center for Cancer Research National Cancer Institute Frederick Maryland USA; ^2^ Metabolic Diseases Branch National Institute of Diabetes and Digestive and Kidney Diseases, National Institutes of Health Bethesda Maryland USA; ^3^ Developmental Therapeutics Branch, Center for Cancer Research National Cancer Institute, National Institutes of Health Bethesda Maryland USA; ^4^ Present address: Cardiovascular Renal and Metabolism BioPharmaceuticals R&D, AstraZeneca Gaithersburg Maryland USA

**Keywords:** MEN1, metformin, pancreatic neuroendocrine tumors, PNETs, tumor prevention

## Abstract

Metformin is a widely prescribed medication in the management of type 2 diabetes mellitus, and numerous epidemiological studies have indicated an association between metformin use and a reduced risk of certain cancers in diabetic populations. However, evidence supporting a role for metformin in cancer prevention remains inconclusive. Exploring tumor‐preventive strategies may be particularly relevant for inherited cancer syndromes with high tumor penetrance such as pancreatic neuroendocrine tumors (PNETs) associated with multiple endocrine neoplasia type 1 (MEN1). MEN1 is caused by germline pathogenic variants in the tumor suppressor gene *MEN1*, and mouse models with *Men1* gene loss also develop PNETs. To evaluate whether metformin is associated with altered PNET outcomes in MEN1, we performed a retrospective analysis of MEN1 patients with PNETs, incorporating detailed clinical exposure data and complemented this analysis with a preclinical study using pancreatic islet β‐cell‐specific *Men1*‐knockout mice treated with metformin. In the clinical cohort, metformin exposure varied substantially with respect to timing and duration relative to PNET diagnosis. No statistically significant association between metformin use and overall survival or metastatic disease was detected in this limited cohort. In the mouse model, treatment with 2 mg/mL metformin in drinking water initiated post‐weaning did not prevent PNET development, nor did it alter circulating insulin or glucose levels. These findings indicate that, under the dosing and exposure conditions examined, metformin was not associated with measurable tumor‐protective or tumor‐preventive effects in MEN1‐related PNETs. The study highlights important methodological considerations, including exposure timing and statistical power, and supports the need for prospective studies initiating metformin prior to tumor development in genetically predisposed populations.

## 1 INTRODUCTION

Metformin is a widely prescribed medication for the management of type 2 diabetes mellitus, and various epidemiological studies have indicated an association between metformin use and a decreased risk of certain cancers.^1^, ^2^ Ongoing clinical trials have been examining its efficacy as a monotherapy or in combination with other anti‐cancer agents for the treatment of cancers such as bladder, breast, colorectal, endometrial, gastric, pancreatic, and prostate.^3^, ^4^ However, current evidence regarding the role of metformin in cancer prevention remains limited.

Investigating the potential of metformin to prevent tumor development could offer significant benefits, particularly for patients with inherited syndromes characterized by high penetrance of specific tumors, such as pancreatic neuroendocrine tumors (PNETs) associated with multiple endocrine neoplasia type 1 (MEN1).^5^, ^6^, ^7^ PNETs can develop sporadically or as a part of hereditary tumor syndromes like MEN1, which is caused by germline heterozygous inactivating pathogenic variants in the *MEN1* gene.^8^ Notably, somatic mutations in the *MEN1* gene have also been identified in 30%–40% of sporadic PNET cases.^9^ Also, PNETs develop in mouse models with targeted deletion of the *Men1* gene and have been instrumental in studying PNET development and testing therapeutic approaches.^10^, ^11^, ^12^

Furthermore, similar to the observed reduction in various cancer risks among diabetic patients taking metformin, retrospective analyses have suggested a protective effect of metformin in patients with sporadic PNETs undergoing standard of care chemotherapy treatment. One such study included 445 patients with PNETs treated across 24 medical centers in Italy; among these patients, those with diabetes taking metformin exhibited significantly longer progression‐free survival (PFS) (median of 44.2 months) compared to diabetic patients on other anti‐diabetic medications (median of 20.8 months).^13^ Multiple clinical trials are currently underway to investigate the anti‐neoplastic effects of metformin in PNETs, both alone and in combination with standard chemotherapy regimens (see www.clinicaltrials.gov; NCT02279758, NCT02294006, and NCT02823691). In vitro studies using neuroendocrine tumor cell lines (QGP‐1 and BON‐1) have shown that metformin can inhibit cell proliferation and migration, further supporting its potential role in PNET management and prevention.^14^, ^15^, ^16^, ^17^, ^18^

Based on these findings, we conducted a retrospective chart analysis of MEN1 patients to assess the association between metformin use and PNET development. Additionally, we initiated a preclinical tumor prevention study with metformin using a mouse model of *Men1* loss.

## 2 METHODS

### 2.1 Retrospective data analysis of MEN1 patients with PNETs

Written informed consent was obtained from all participants included in this analysis prior to enrollment, in accordance with the protocols approved by the Institutional Review Board for the Natural History Study of Parathyroid Disorders (ClinicalTrials.gov Identifier: NCT04969926) conducted at the NIH Clinical Center in Bethesda, Maryland. Data pertaining to patients with PNETs with confirmed germline *MEN1* pathogenic variants, evaluated at our institution under this protocol between 1979 and 2019, were extracted from the institution's secure database. Clinical variables collected included age at metformin initiation, sex, timing of metformin exposure relative to PNET diagnosis (before, concurrent with, or after diagnosis), duration of metformin use, disease extent (localized vs. metastatic), multifocality, and history of pancreatic surgery. Timing and duration of metformin exposure varied widely across patients, introducing potential immortal‐time bias. Therefore, survival analyses were interpreted descriptively, and time‐dependent Cox proportional hazards models were used where feasible to account for delayed treatment initiation.

### 2.2 Animals and treatment

All animal experiments were performed according to the animal study protocols approved by the Institutional Animal Care and Use Committee of NIDDK (Animal Study Protocol number: K070‐MDB‐24), and National Cancer Institute (NCI)‐Frederick Animal Care and Use Committee (NCI‐Center for Advanced Preclinical Research Animal Study Protocol number: 17‐044). The generation, genotyping, and characterization of mouse lines with pancreatic islet β‐cell‐specific Cre expression driven by a ~670 bp fragment of the rat insulin II gene promoter (RIP‐Cre) in the C57BL/6 background (RIP‐Cre mice), and a conditional pancreatic islet β‐cell‐specific knock‐out of *Men1* (RIP‐Cre;*Men1* ^f/f^) in the mixed 129Sv/FVB and C57BL/6 background was previously described.^19^ In the RIP‐Cre;*Men1* ^f/f^ mouse line, pituitary tumors are a significant cause of mortality, seen in 81% of females and 23% of males.^19^ Therefore, to avoid premature death of female mice due to pituitary tumors, only male mice were used in this study. Five mice were housed per cage and were given standard mouse maintenance diet (PMI CRL Rat and Mouse Diet, Cat. #RHI5L79, Animal Specialties and Provisions).

At the time of weaning (18–21 days after birth), male RIP‐Cre;*Men1* ^f/f^ mice were randomized into four arms of the study: groups 1 and 3 were given water bottles with normal (purified by reverse osmosis pre‐filtered) animal facility water and groups 2 and 4 were given water bottles with 2 mg/mL metformin in the drinking water for ad libitum drug intake. Water bottles were dated and prepared fresh weekly. When a new water bottle was placed in the cage, the water remaining in the previous bottle was measured to monitor water consumed weekly (similar volumes were consumed from bottles with or without metformin). Mice were weighed and observed weekly. At 3–4 months post‐weaning, groups 1 and 2 were assessed for the effect of short‐term metformin treatment. At 10–11 months post‐weaning, groups 3 and 4 were assessed for the effect of long‐term metformin treatment. As a control, male wild type mice (RIP‐Cre) (*n* = 10) were maintained on normal drinking water to follow the increase in islet size of RIP‐Cre;*Men1* ^f/f^ mice.

Metformin was purchased from the NIH Division of Veterinary Research (Metformin hydrochloride USP, 500 mg tablets, manufactured by Granules India Limited) and dissolved in purified by reverse osmosis pre‐filtered animal facility water to a final concentration of 2 mg/mL. The amount of metformin in the drinking water that was used in our study is based on the dose to achieve clinically relevant plasma concentration in humans and experiments conducted in mice, and the daily water intake confirmed in our animal colony to be on the average 4 mL/mouse.^20^, ^21^, ^22^ Therefore, further assessment of plasma metformin levels was not performed in our study.

### 2.3 Pancreas histology and measurement of islets

Pancreas was collected in 10% buffered formalin and after 48 h transferred to 70% ethanol. Formalin fixed and paraffin embedded (FFPE) pancreas tissue blocks were prepared and 5 μm sections were used for hematoxylin and eosin (H&E), and slides were imaged at 20× using digital slide scanning software (Aperio AT2 ImageScope Viewing and Acquisition package, Leica Biosystems). Slides were evaluated by a board‐certified veterinary pathologist to observe tissue histology and perform quantitative assessment of islets. The maximum diameter of individual islets was measured, and an AI classifier (Halo Densenet v2, Indica Lab) was used to segment pancreatic tissue into exocrine and endocrine compartments for quantifying the percentage of total pancreatic area occupied by islets.

### 2.4 Serum biochemistry

Whole blood was collected in gold top serum separator tubes (BD Microtainer Blood Collection Tubes, Becton Dickinson, Catalog #365967) to process for serum isolation. The serum was aliquoted in multiple tubes and stored at −80°C. Insulin and glucose were assayed in the serum samples with the following kits: Ultra‐Sensitive Mouse Insulin ELISA Kit (Crystal Chem, Catalog #90080) and Mouse Glucose Assay Kit (Crystal Chem, Catalog #81692).

### 2.5 Statistics

Statistical analyses were performed using GraphPad Prism software (version 10). Overall survival in the clinical cohort was analyzed using Kaplan–Meier survival curves with log‐rank testing. For animal studies, comparisons of continuous variables between two groups were performed using Student's *t*‐tests.

Missing clinical data were handled through predefined exclusion criteria, including exclusion of patients without confirmed PNET diagnosis or without a minimum of 5 years of follow‐up. Due to the limited number of MEN1 patients exposed to metformin and the small number of outcome events, multivariable analyses were not statistically robust and therefore were not performed. This limitation was taken into account in the interpretation of results.

## 3 RESULTS

### 3.1 Baseline characteristics of MEN1 patients with and without metformin exposure

Among the 110 MEN1 patients with confirmed PNETs included in the analysis, 16 had documented exposure to metformin. Baseline characteristics of patients with and without metformin exposure are summarized in Table 1. Patients in the metformin group were similar to those not receiving metformin with respect to sex distribution and frequency of metastatic disease. The mean age at metformin initiation was 53 years (median 54; range 25–71 years).

**TABLE 1:** Baseline characteristics of MEN1 patients with pancreatic neuroendocrine tumors.

Characteristic	Metformin (*n* = 16)	No metformin (*n* = 94)
Female (%)	62.5	53
Mean age at metformin start (years)	53 ± 13	—
Metastatic disease (%)	50	45
Locally advanced disease (%)	31	19
Multifocal disease (%)	13	35
Prior pancreatic surgery (%)	63	46

Metformin exposure occurred heterogeneously relative to PNET diagnosis. Among the 16 MEN1 patients treated with metformin, four initiated metformin prior to PNET diagnosis (ranging from 2 to 11 years earlier), two initiated metformin in the same year as PNET diagnosis, and 10 initiated metformin after diagnosis (ranging from 1 to 28 years later). The duration of metformin use varied substantially across patients, with a median duration of 2 years and a mean duration of 5 years.

Detailed measures of glycemic control, including hemoglobin A1c levels, as well as other metabolic or cardiovascular prognostic factors such as hypertension and dyslipidemia, were not consistently available across the cohort and therefore could not be reliably analyzed. Given these data limitations and the small number of metformin‐exposed patients, disease‐specific survival and progression‐free survival could not be robustly assessed. Accordingly, analyses were limited to overall survival and metastatic status, and the results are interpreted descriptively.

**FIGURE 1:**
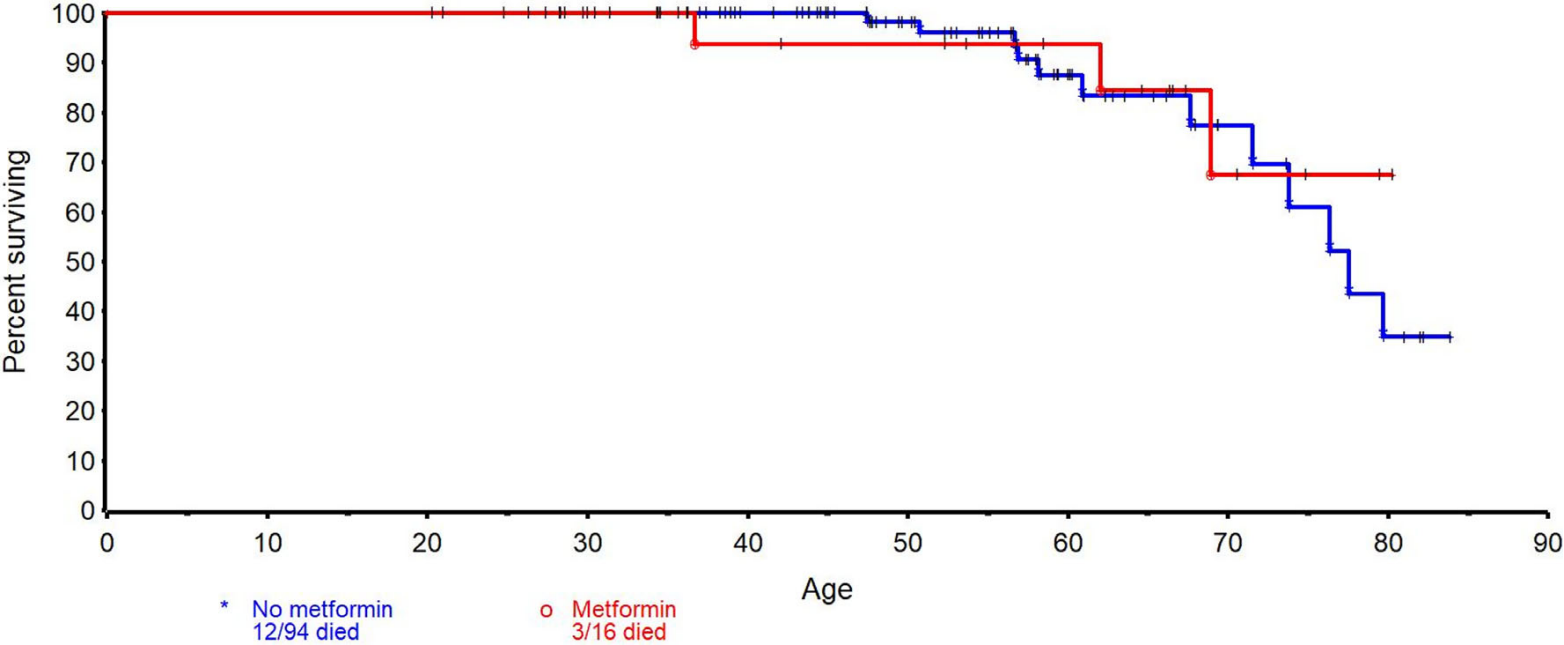
Effect of metformin use in MEN1 patients with PNETs. Kaplan–Meier analysis showing overlapping survival curves between groups, MEN1 patients with PNETs taking (red) or not taking (blue) metformin. No statistically significant association between metformin use and overall survival was detected.

Kaplan–Meier analysis demonstrated overlapping survival curves between groups. (Figure 1). Time‐dependent Cox regression analysis yielded a hazard ratio of 1.20 (95% CI 0.27–3.96; *p* = .79), although confidence intervals were wide due to the limited number of exposed patients and events. In this retrospective cohort, the number of MEN1 patients exposed to metformin was limited (*n* = 16). No statistically significant association between metformin use and overall survival or metastatic disease was observed. Given the small sample size and heterogeneity in the timing and duration of metformin exposure, these analyses were contexts for exploratory interpretation only.

### 3.2 Effect of metformin treatment on PNET development in *Men1*‐knockout mice

Mice with conditional homozygous *Men1* knockout in the pancreatic islet β‐cells (RIP‐Cre;*Men1* ^f/f^) develop islet tumors (PNETs) with 100% penetrance.^19^, ^23^, ^24^ These PNETs arise from the islet β‐cells (insulinoma) and therefore they over‐secrete insulin that can be detected in the blood. Compared to the conventional *Men1*‐knockout mice (*Men1* ^+/−^), the RIP‐Cre;*Men1* ^f/f^ mice start developing islet hyperplasia (age >2 months) and islet tumors (age >9 months) at an early age because they do not depend upon the spontaneous somatic loss of the second copy of the *Men1* gene for tumor formation.^19^, ^23^, ^24^, ^25^, ^26^, ^27^, ^28^ To assess the effect of metformin, we used the RIP‐Cre;*Men1* ^f/f^ mice^19^ that show 100% penetrance for PNETs and at an early age, which reduced the duration of this study to detect the efficacy of metformin in the prevention of PNETs.

**FIGURE 2:**
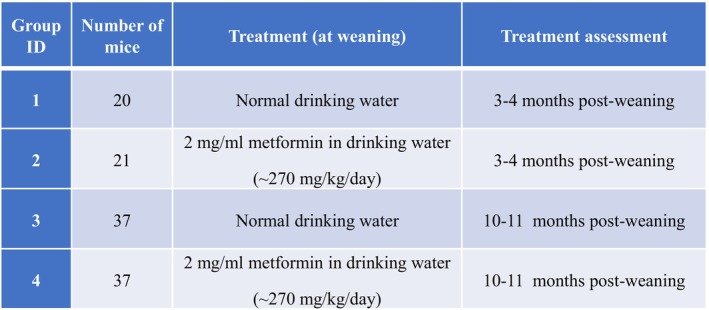
Study design of metformin treatment in a mouse model with MEN1‐associated PNETs. Starting at the time of weaning, male RIP‐Cre;*Men1* ^f/f^ mice were given normal drinking water or 2 mg/mL metformin in drinking water. The cage water bottles were changed weekly. After 3–4 months post‐weaning, blood and pancreas were collected from groups 1 and 2. After 10–11 months post‐weaning, blood and pancreas were collected from groups 3 and 4.

A cohort of 116 male RIP‐Cre;*Men1* ^f/f^ mice were established and randomized into two groups. At the time of weaning (18–21 days after birth), 58 mice were provided ad libitum with normal drinking water and 58 mice with metformin (at 2 mg/mL) in the drinking water, with a weekly change of the freshly prepared water bottles (Figure 2). The effect of metformin was assessed after 3–4 months and 10–11 months post‐weaning, early and late point, respectively. WT mice (RIP‐Cre) served as non–tumor‐prone controls that were provided normal drinking water.

### 3.3 Effect of metformin on body weight and mortality of *Men1*‐knockout mice

**FIGURE 3:**
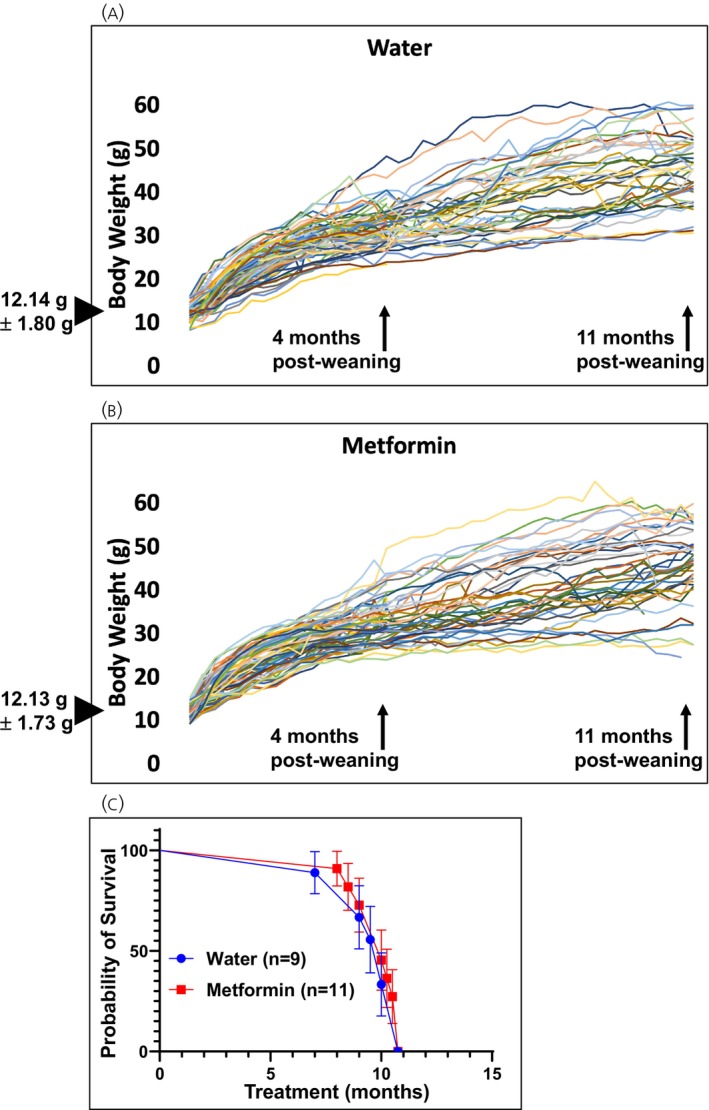
Body weight and mortality of RIP‐Cre;*Men1* ^f/f^ mice given normal drinking water or 2 mg/mL metformin in drinking water post‐weaning. (A, B) Body weight of mice on normal drinking water or metformin‐containing water (up to study endpoint). Mean body weight at the start of the study is shown on the left. Metformin treatment did not affect body weight (*p*‐value >.1). (C) Metformin treatment did not affect the mortality of mice during the study (*p*‐value >.1).

No significant differences in body weight were observed between RIP‐Cre;*Men1* ^f/f^ mice receiving metformin‐supplemented drinking water and those receiving normal drinking water. (Figure 3A,B). Similarly, metformin treatment did not affect overall survival during the study period (Figure 3C). Therefore, metformin treatment did not affect the body weight or mortality of *Men1*‐knockout mice.

### 3.4 Effect of metformin on tumor burden and pancreatic histology in *Men1*‐knockout mice

**FIGURE 4:**
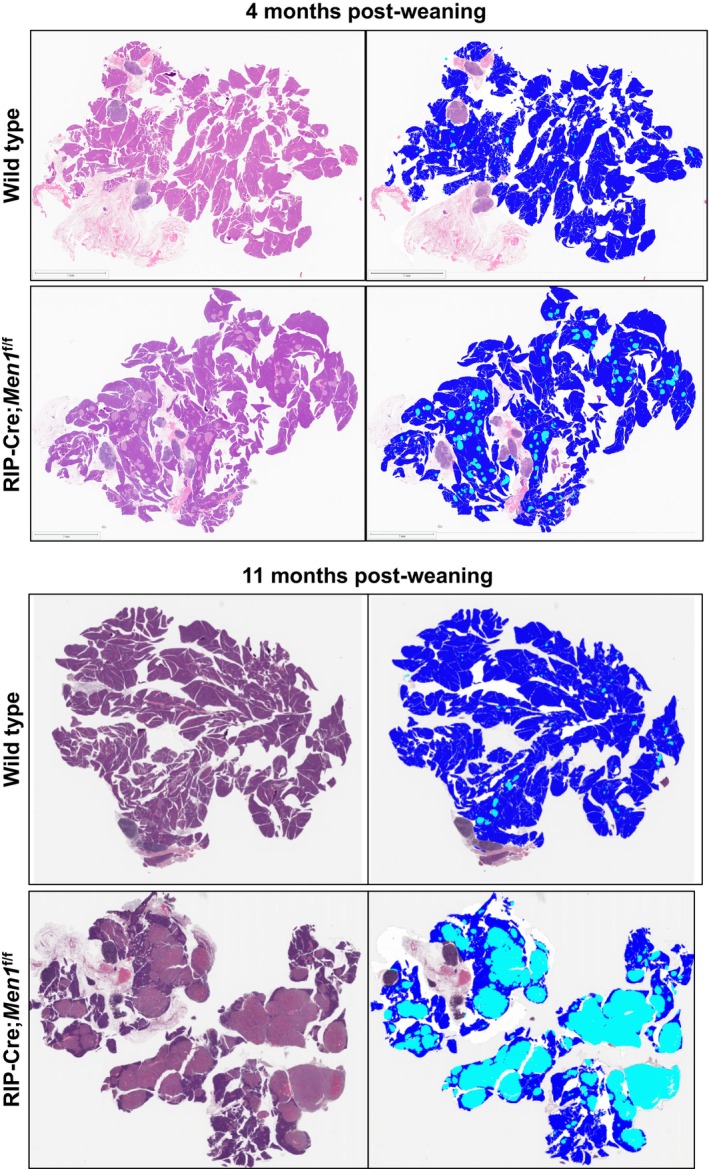
Islet size measurement. Representative microscopy images of FFPE pancreas sections, H&E‐stained (left) and islet size quantification pseudo‐colored (right) showing wild type normal islets (4 months and 11 months post‐weaning) and RIP‐Cre;*Men1* ^f/f^ islet hyperplasia (4 months post‐weaning) and islet tumors (11 months post‐weaning). Pseudo‐colors used for quantification of islet size in Halo Densenet v2 image analysis software, exocrine (blue) and endocrine/islets (turquoise).

**FIGURE 5:**
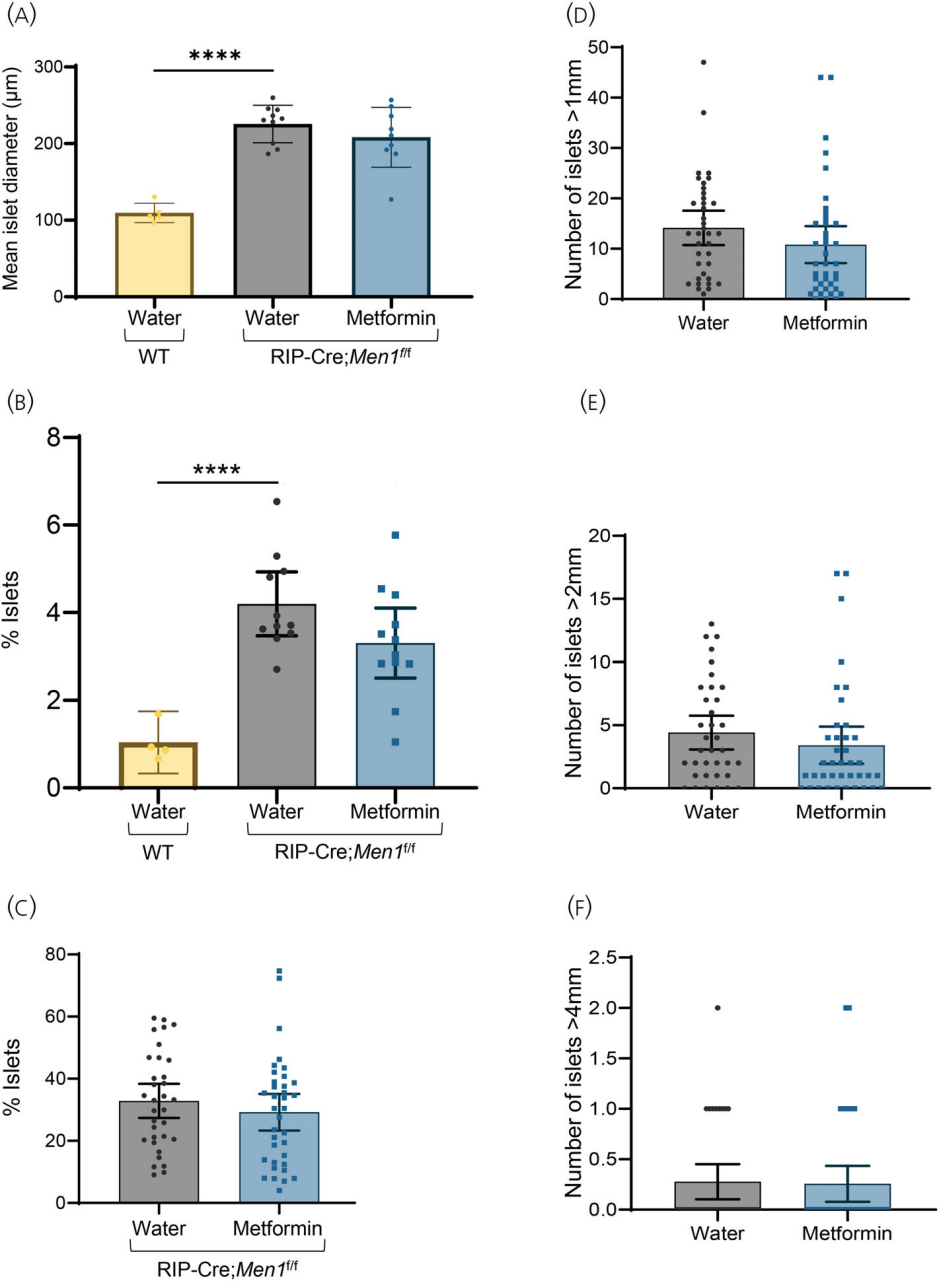
Islet size of RIP‐Cre;*Men1* ^f/f^ mice given normal drinking water or 2 mg/mL metformin in drinking water post‐weaning. (A) Islet size (diameter) after 3–4 months post‐weaning was significantly increased in RIP‐Cre;*Men1* ^f/f^ mice compared to WT (****p‐value <.01). Metformin treatment did not affect islet size of RIP‐Cre;*Men1* ^f/f^ mice (*p*‐value >.1). (B) Percentage of islets in the pancreas after 3–4 months post‐weaning was significantly increased in RIP‐Cre;*Men1* ^f/f^ mice compared to WT (****p‐value <.01). Metformin treatment did not affect the percentage of islets in the pancreas (*p*‐value >.1). (C) Treatment with metformin for 10–11 months post‐weaning did not affect the percentage of islets in the pancreas of RIP‐Cre;*Men1* ^f/f^ mice (*p*‐value >.1). (D–F) Treatment with metformin for 10–11 months post‐weaning did not affect the growth of tumors as indicated by the number of islets >1, >2, or >4 mm in RIP‐Cre;*Men1* ^f/f^ mice (*p*‐value >.1).

As expected, compared to WT mice, RIP‐Cre;*Men1* ^f/f^ mice exhibited pancreatic islet hyperplasia at 4 months post‐weaning and developed islet tumors (PNETs) by 10–11 months post‐weaning (Figure 4). Quantitative histological analysis demonstrated increased islet size and islet area in RIP‐Cre;*Men1* ^f/f^ mice relative to WT controls (Figure 5A,B). However, no significant differences in islet diameter, percentage of pancreatic area occupied by islets, or number of large islets were observed between RIP‐Cre;*Men1* ^f/f^ mice on normal drinking water or metformin‐supplemented drinking water at either early or late time points (Figure 5A–F). Therefore, metformin treatment did not affect pancreatic islet hyperplasia or PNET development and growth in *Men1*‐knockout mice.

### 3.5 Effect of metformin on circulating insulin and glucose in *Men1*‐knockout mice

**FIGURE 6:**
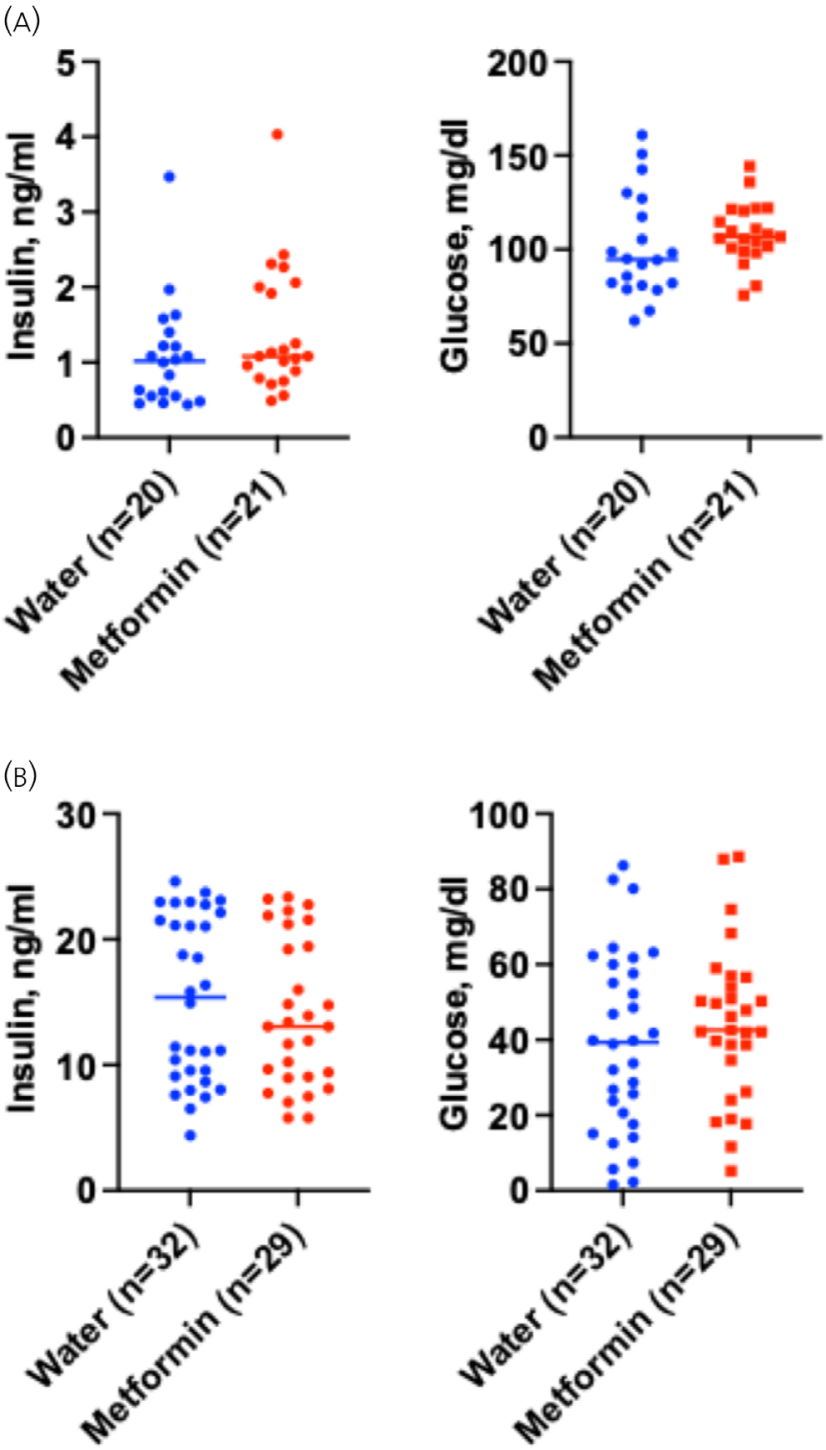
Serum biochemistry of RIP‐Cre;*Men1* ^f/f^ mice given normal drinking water or 2 mg/mL metformin in drinking water post‐weaning. (A) Metformin treatment for 3–4 months post‐weaning did not affect serum insulin or glucose (*p*‐value >.1). The number of mice in each group is shown in parentheses. (B) Metformin treatment for 10–11 months post‐weaning did not affect serum insulin or glucose (*p*‐value >.1). The number of mice in each group is shown in parentheses.

PNET lesions in RIP‐Cre;*Men1* ^f/f^ mice over‐secrete insulin as they arise from the islet β‐cells (insulinoma).^19^, ^23^, ^24^ Therefore, these mice show increased circulating levels of insulin that reduce glucose levels. Metformin treatment did not significantly alter serum insulin or glucose concentrations at either early or late time points (Figure 6A,B).

## 4 DISCUSSION

Metformin treatment has been associated with a reduced incidence of cancer in epidemiological studies of patients with type 2 diabetes^1^, ^2^, ^3^, ^4^ and retrospective analyses in patients with sporadic PNETs have suggested improved progression‐free survival among diabetic patients receiving standard‐of‐care therapies concurrently treated with metformin.^13^ These observations, together with the growing body of preclinical data implicating metformin in the modulation of cellular metabolism and growth signaling pathways, provided the rationale for investigating whether metformin might exert a tumor‐protective or tumor‐preventive effect in individuals with a germline predisposition to PNETs, such as those with MEN1.

MEN1 is characterized by germline inactivating pathogenic variants in the *MEN1* tumor suppressor gene, leading to loss of function of its encoded protein menin and a high lifetime risk of developing multiple endocrine tumors, including PNETs in up to 80% of patients.^6^, ^7^ Given this high penetrance and the often multifocal and recurrent nature of PNETs in MEN1, even relatively modest protective or preventative effects could have meaningful clinical implications. Therefore, we investigated whether metformin use, as documented within our MEN1 patient cohort, was associated with differences in PNET occurrence or metastatic progression. In this well‐characterized clinical population, we did not observe a statistically significant reduction in the presence of PNETs or their metastases among patients taking metformin compared to those not on the medication.

From a methodological perspective, it is important to interpret these negative findings considering several limitations inherent to our retrospective analysis. Interpretation of the clinical findings is limited by the small number of MEN1 patients treated with metformin (*n* = 16), which substantially restricts statistical power. Interpretation of the clinical findings is further limited by incomplete information regarding metformin dosing. While the timing and duration of metformin exposure could be summarized descriptively, precise dose data were not consistently documented in this retrospective cohort, precluding dose–response analyses. Interpretation is further constrained by heterogeneity in the timing and duration of metformin exposure, as most patients initiated therapy after PNET diagnosis rather than prior to tumor development. In addition, incomplete availability of glycemic control metrics and other potential prognostic variables precluded adjustment for metabolic or cardiovascular confounders. Together, these limitations restricted the ability to robustly evaluate disease‐specific survival or progression‐free survival and limit causal inference inherent to the retrospective study design. Under the conditions examined, no statistically significant association was detected in this limited cohort, and these findings should not be interpreted as evidence of absence of effect. Larger, prospectively designed studies with standardized exposure timing and comprehensive metabolic characterization will be required to more definitively evaluate the potential role of metformin in MEN1‐associated PNETs.

To corroborate the clinical observations, we conducted a complementary controlled preclinical study using a pancreatic islet β‐cell‐specific *Men1*‐knockout mouse model that recapitulates key aspects of MEN1‐associated PNET tumorigenesis. This model develops insulin‐secreting functional PNETs (insulinomas) with complete penetrance and allows longitudinal assessment of tumor development in a genetically predisposed background.^19^ This model offers two distinct advantages: first, *Men1* deletion occurs within a defined cell type, paralleling the origin of many MEN1‐associated PNETs; second, tumor development proceeds spontaneously in a background that closely resembles inherited predisposition, without the influence of environmental carcinogens or transplantation procedures. These features enable a more relevant evaluation of metformin's potential preventive effects in a setting that emulates human disease. It should be noted, however, that the mouse model used in this study evaluates the development of insulin‐secreting functional PNETs (insulinomas) and does not capture the full spectrum of non‐functioning PNETs observed in MEN1 patients.

In our study, only a single dose of provided ad libitum metformin (administered as a 2 mg/mL metformin solution in drinking water) was evaluated. Although plasma metformin concentrations were not directly measured in our experiments, weekly water consumption was monitored throughout the study. The selected dose was based on prior published studies demonstrating clinically relevant systemic exposure in mice subjected to treatment with the dose selected for our experiments or lower metformin doses.^29^ Furthermore, in previous studies, it has been demonstrated that following a similar route of metformin administration—ad libitum in drinking water formulated with 1.25 mg/mL metformin, corresponding to an effective daily oral uptake of 250 mg/kg—resulted in systemic drug concentrations reaching 5 and 40 μM in vivo in circulating plasma and liver tissue, respectively, representing a 10‐fold higher dose of the drug versus the one given to human patients subject to routine diabetes treatment, on a mg/kg basis.^20^ While we have considered conducting similar experiments using higher dose levels of metformin, for example, up to 5 mg/mL in drinking water, concerns of significantly surpassing the clinically relevant doses of metformin, as well as prior reports of systemic metformin toxicity in experimental rodent models at doses exceeding 500 mg/kg discouraged us from conducting experiments at elevated drug loads.^30^

We assessed multiple endpoints relevant to tumor prevention and found that age at tumor onset and progression were comparable between metformin‐treated and untreated *Men1*‐knockout mice, while no significant differences in circulating insulin or glucose levels were observed at either early or late stages. These findings indicate that, under the dosing and timing conditions examined, metformin did not produce measurable effects on tumor development or metabolic parameters, and no evidence was found to support an indirect anti‐tumor effect mediated through alterations in circulating metabolic hormones. Furthermore, the absence of direct pharmacokinetic measurements limits precise correlation between administered dose and circulating drug levels and precludes assessment of potential dose–response effects.

When interpreting these preclinical results, several methodological considerations should be highlighted. First, only a single dose of metformin was tested; although 2 mg/mL is commonly used in mouse chemoprevention protocols, it remains possible that this dose was insufficient to impact β‐cell or tumor metabolism in the context of *Men1* deficiency, especially if higher intratumoral concentrations are necessary to activate pathways such as AMPK or inhibit mTOR signaling.^22^ Second, the initiation of treatment occurred post‐weaning at 3 weeks of age, driven by practical considerations related to drug administration. However, in this model, *Men1* deletion occurs during embryogenesis (from approximately day 13.5 of gestation), suggesting that early oncogenic events likely occurred prior to the start of metformin exposure. If metformin primarily acts to prevent tumor initiation rather than progression, initiating treatment after early genetic events could limit its efficacy, an important consideration for designing future interventions.

Despite these limitations, the study design offers valuable insights. Because tumors develop spontaneously in a genetically predisposed background, longitudinal assessment of tumor emergence and progression can be performed without confounding factors associated with artificial tumor induction methods. The absence of a detectable effect under these experimental conditions suggests that, at the dose and timing used, metformin alone may not be sufficient to significantly alter PNET development driven by *Men1* loss in β‐cells. Nevertheless, these data generate testable hypotheses for future work and further studies with metformin use in pregnant mice and/or a higher dose of metformin could be informative to determine the potential of metformin in preventing or delaying PNET development in this mouse model.

In summary, integration of detailed clinical exposure data with preclinical modeling did not identify a measurable association between metformin use and PNET outcomes in MEN1 under the specific exposure and drug dosing conditions studied. Of note, while our study was under review, Nakano et al. reported that long‐term treatment with a supratherapeutic dose of metformin (5 mg/mL) in drinking water in a different *Men1*‐knockout model (that deletes *Men1* exons 3–6 in the pancreatic islet β‐cells as opposed to the mouse model used in our studies featuring deletion of exons 3–8) appeared to suppress PNET progression, indicating a potential preventive effect.^19^, ^31^, ^32^ Collectively, these findings underscore the importance of model choice, exposure timing, choice of therapeutic dose, selection of informative outcomes, and the overall study design in evaluating tumor‐preventive strategies and provide a framework for future prospective investigations.

## AUTHOR CONTRIBUTIONS

**Serguei V. Kozlov:** Conceptualization; data curation; formal analysis; investigation; methodology; project administration; resources; supervision; validation; visualization; writing – review & editing. **Sunita K. Agarwal:** Conceptualization; data curation; formal analysis; investigation; methodology; project administration; resources; supervision; visualization; writing – original draft; writing – review & editing. **Theresa M. Guerin:** Data curation; investigation; methodology; resources; validation. **Wendi Custer Lawrence:** Investigation; methodology; resources. **Laura L. Bassel:** Data curation; methodology; resources; investigation; visualization; writing – review & editing. **Akua Graf:** Data curation; writing – review & editing. **Smita Jha:** Resources; writing – review & editing. **Lee S. Weinstein:** Resources; writing – review & editing. **Jenny E. Blau:** Resources; writing – review & editing. **Jaydira del Rivero:** Conceptualization; data curation; formal analysis; investigation; methodology; project administration; resources; visualization; writing – review & editing.

## FUNDING INFORMATION

This work was supported by the Intramural Research Program of the National Institutes of Health: National Institute of Diabetes and Digestive and Kidney Diseases grant number 1ZIADK075035 (S.K.A.) and 1ZIADK043006 (S.J.), National Cancer Institute grant number Z01‐BC 006150 (J.D.R.), and in part with federal funds from the National Cancer Institute under the Contract number HHSN261200800001E.

## CONFLICT OF INTEREST STATEMENT

The authors declare no conflicts of interest.

## ETHICS APPROVAL

All animal experiments were performed according to the animal study protocols approved by the National Institute of Diabetes and Digestive and Kidney Diseases (NIDDK) Institutional Animal Care and Use Committee (NIDDK Animal Study Protocol number: K070‐MDB‐24) and by the National Cancer Institute (NCI)‐Frederick Animal Care and Use Committee (NCI‐Center for Advanced Preclinical Research Animal Study Protocol number: 17‐044).

## PATIENT CONSENT

Written informed consent was obtained from all patients under IRB‐approved “Natural History Study of Parathyroid Disorders” (NCT04969926) conducted at the National Institutes of Health (NIH) Clinical Center, Bethesda, Maryland.

## Data Availability

All the mouse‐related data are presented in this report. For human data sharing, evaluation of the request will be done by the local Research Ethics Board and the signing of a data transfer agreement.
